# One Health integrated surveillance: a way forward to accelerate schistosomiasis elimination in China

**DOI:** 10.1016/j.soh.2025.100114

**Published:** 2025-05-16

**Authors:** Suying Guo, Lijuan Zhang, Yifeng Li, Shiqing Zhang, Xiaojuan Xu, Yinlong Li, Chunli Cao, Jing Xu, Shizhu Li

**Affiliations:** a National Institute of Parasitic Diseases, Chinese Center for Disease Control and Prevention (Chinese Center for Tropical Diseases Research), National Key Laboratory of Intelligent Tracking and Forecasting for Infectious Diseases, Key Laboratory on Parasite and Vector Biology, Ministry of Health, WHO Centre for Tropical Diseases, National Center for International Research on Tropical Diseases, Ministry of Science and Technology, Shanghai 200025, China; b Jiangxi Provincial Institute of Parasitic Diseases, Nanchang 330096, Jiangxi, China; c Anhui Provincial Center for Disease Control and Prevention, Anhui Provincial Academy of Preventive Medicine, Anhui Provincial Institute of Schistosomiasis Control, Hefei 230601, Anhui, China; d School of Global Health, Chinese Center for Tropical Diseases Research, Shanghai Jiao Tong University School of Medicine, Shanghai 200025, China

**Keywords:** One Health, Surveillance, Schistosomiasis, Elimination

## Abstract

Surveillance is an effective approach for disease control and prevention. Being a vector-borne and zoonotic parasitic disease, schistosomiasis has been under comprehensive surveillance in China for several decades, with focus on indicators related to definitive hosts, intermediate hosts, as well as changes of influencing factors. This article reviewed the surveillance system of schistosomiasis in China from the perspective of One Health to provide evidence for the acceleration of elimination. When moving towards elimination with rare new infection occurred in humans, livestock and snails, One Health surveillance system could be the most effective approach to accelerate the process of elimination or consolidate the achievement of schistosomiasis by integrating the risk surveillance and novel diagnostic tools in the intelligent multi-point trigger infectious disease monitoring and early warning system.

## 1 Background

Schistosomiasis is one of the most important neglected tropical diseases and a severe issue of public health in the world [1]. Schistosomiasis affects more than 240 million people worldwide, and more than 700 million people live in the danger of infection in endemic areas [2]. In China, only schistosomiasis japonica, caused by *Schistosoma japonicum*, is prevalent along the Yangtze River and its south region [3]. There were 11.6 million patients (about 600,000 were advanced cases), 1.2 million infected cattle, and 14.3 billion square meters of infested area with *Oncomalania hupensis* in the mid-1950s [4]. After several decades of implementation of consecutive control activities, China is moving towards the elimination of schistosomiasis.

Surveillance is an effective approach to detect emerging cases, identify the current status of prevalence and transmission risk, and provide guidance to implement appropriate interventions [5]. Timely dissemination of surveillance results can improve the planning, implementation, and evaluation of public health practice [6]. There is an urgent need to improve the surveillance and response system for schistosomiasis to prevent disease rebound or reintroduction, as the trend of prevalence varies from pretty high to low among humans and livestock, and the targets set for schistosomiasis shifted from transmission control to elimination in China. In this article, we describe the concept of One Health surveillance briefly, review the evolution of the schistosomiasis surveillance system, particularly focusing on sentinel surveillance in China, and discuss the application of the One Health approach in the surveillance response system to advance the process of schistosomiasis elimination in China.

## 2 Search strategy and selection criteria

References for this review were identified through searches of PubMed, Web of Science, Science Direct, and China National Knowledge Infrastructure (CNKI) for articles published from January 2000 to June 2024 by use of the terms “schistosomiasis,” “schistosome,” and “surveillance.” Articles resulting from these searches and relevant references cited in those articles were reviewed. Articles published in English and Chinese were included. Besides, five versions of the National Schistosomiasis Surveillance Plan were also collected to compare the changes in the main content of the plans.

## 3 One Health surveillance

The “One Health” concept derives from the “One Medicine” of the combination of human medicine and veterinary medicine and then the “One World–One Health” concept, which calls for another incorporation of the eco-system health [7,8]. According to the definition by the Food and Agriculture Organization of the United Nations, the World Organisation for Animal Health, the United Nations Environment Programme, and the World Health Organization, One Health is an integrated, unifying approach that aims to sustainably balance and optimize the health of people, animals, and ecosystems [9]. It encourages and expands multi-disciplinary collaborations, integrative research, capacity building, clinical practice, policy, and communication among many stakeholders [8,10]. One Health surveillance can be explained as implementing the One Health approach to improve health by collecting data and producing information to support integrated actions across human and animal health and environment sectors [11].

The implementation of the One Health approach was propelled by the catastrophic prevalence of H5N1 in Asia in late 2003, when it became clear that the disease should be understood and controlled by following the entire transmission chain, including the infected individuals and the environment [12]. This concept has been used in the regular surveillance system of Nipah virus [13] and parasitic diseases led by *Toxoplasma gondii*, *Echinococcus granulosus*, *Toxocara canis*, and *Trichinella*, etc. [14], as well as other diseases, including COVID-19 [[15], [16], [17]] and antimicrobial resistance [18]. Indeed, the intention of One Health is not a novel concept to the parasitologists [19] because many parasitic diseases are zoonotic with the control of the intermediate hosts. Although the focus of the surveillance varies in different endemic stages of schistosomiasis from morbidity control to elimination, the One Health concept is always along with the development of schistosomiasis surveillance system, when monitoring every point of the transmission chain, such as the definitive hosts of human and animals, the intermediate hosts of snails, and risk factors such as social and environmental determinants which may affect the transmission of schistosomiasis.

## 4 The general scheme of the schistosomiasis surveillance system in China

The Chinese surveillance for schistosomiasis began in the middle of the 20th century after the founding of the People's Republic of China, with the identification of endemic areas through both hospital-based case reporting and limited epidemiological surveys [20]. Currently, the surveillance system mainly consists of passive surveillance (routine surveillance) based on case reports, repetitive cross-sectional sampling surveys, and longitudinal sentinel surveillance [5].

As schistosomiasis was listed as one of the four top infectious diseases given high priority and up-graded from C catalog to B catalog in the national notifiable infectious disease list of China [21], the passive routine surveillance on schistosomiasis was initiated in 2004, covering 31 provinces/autonomous regions/municipalities (P/A/M) (not including Hong Kong, Macao, and Taiwan) [22]. It demands routine statutory reports of schistosomiasis from subordinate units to the superior units. Routine surveillance includes case reports and confirmations, epidemiological surveys and reports on the confirmed cases, acute schistosomiasis warnings, and reports of emergency public health events indicating an outbreak of schistosomiasis. The case report is required within 24 h after the diagnosis of a schistosomiasis patient, followed by a diagnosis recheck by the local Center for Disease Control and Prevention within another 24 h. For confirmed cases, the epidemiological survey should be completed within seven days after diagnosis, and detailed information should be uploaded to the national information systems within two days [23]. A warning on acute schistosomiasis cases will be triggered once a suspicious or diagnosed acute case is uploaded into the disease reporting system, and all the staff at the provincial, municipal, and county-level institutions who are responsible for schistosomiasis data management will receive a warning text on their cellphones. Once the message is received, professionals should validate and upload the information of the reported case within 2 h. The detection of the outbreak of schistosomiasis should be reported within 2 h to the local health authority, and further response would be carried out by the local government.

To fully understand the endemic situation of schistosomiasis and thus provide reliable and scientific reference to design the national control plan or assess the effects of control strategies, four representative national cross-sectional sampling surveys were conducted in 1989, 1995, 2004, and 2016, respectively [21]. The data revealed the prevalence of schistosomiasis among humans, domestic animals and explored the distribution and infection status of snails in the first three surveys. Particularly, the third cross-sectional survey conducted in 2004 played a pivotal role in guiding the design of the national medium and long-term strategic plan that spanned from 2004 to 2015, which effectively suppressed the rebound of schistosomiasis after the completion of the World Bank Loan Project for schistosomiasis in 2001 and accelerated the process of schistosomiasis control in P.R. China. The fourth survey, which focused on the intermediate snail host, helped to understand the change pattern of snail habitats and produced a national snail distribution map at the scale of the environment for the first time. These four surveys provide strong evidence for the evaluation of the effectiveness of the interventions against schistosomiasis during a certain period and reliable reference for the subsequent policy formulation to the next stage.

**Fig. 1:**
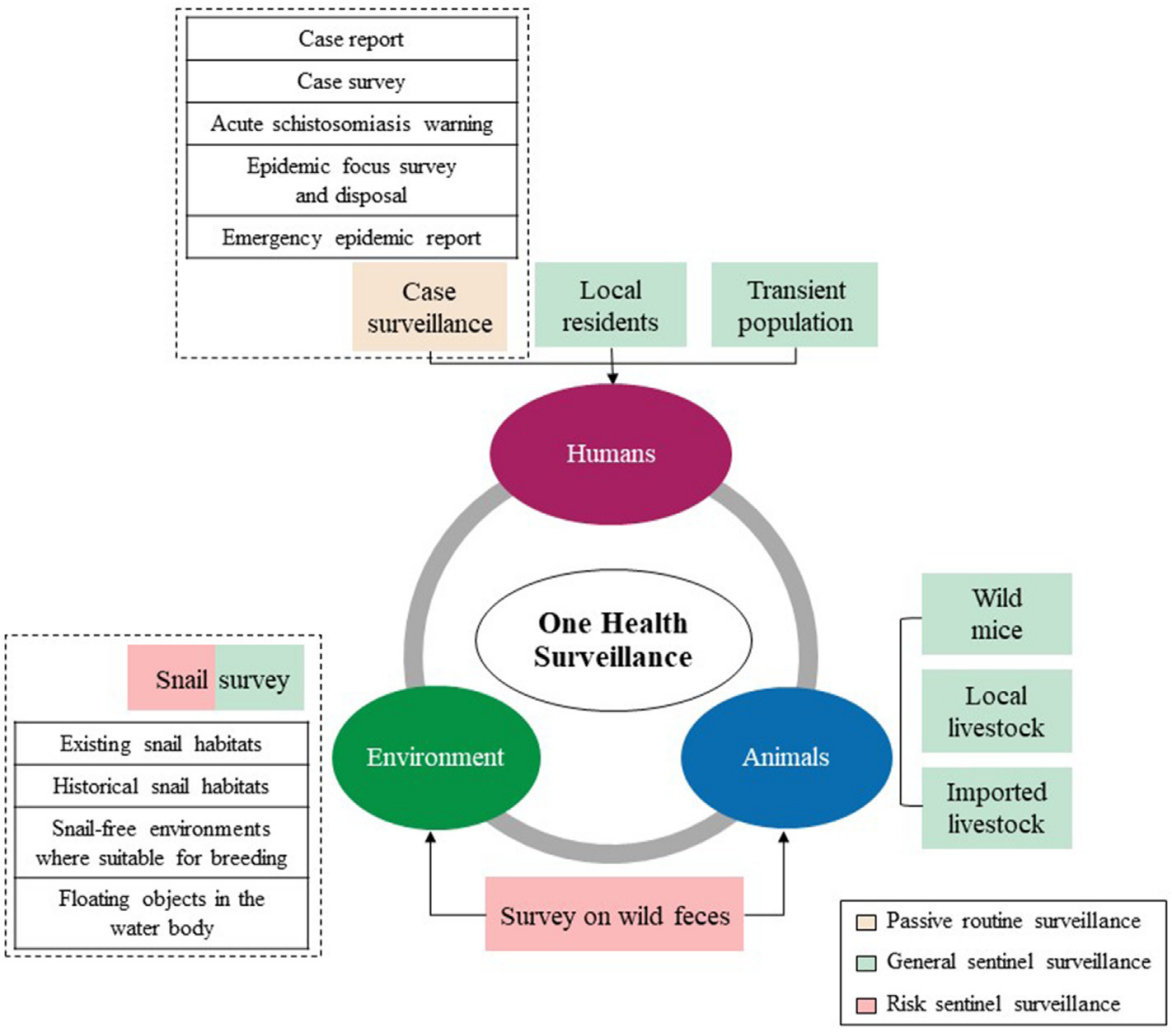
Content of current One Health surveillance on schistosomiasis in China.

Sentinel surveillance is the core of the schistosomiasis surveillance system in China. According to the monitored focus and covered scope, the process of sentinel surveillance for schistosomiasis can be mainly categorized into six stages. The earliest national surveillance based on sentinel sites was initiated in 1990. Along with the reduction of the number of infected residents and livestock, as well as the decrease in snail burden, the foci of surveillance gradually transferred from the infectious resources to the risky environments. To adapt to different prevalence and transmission risks in different stages, five versions of the National Surveillance Plan of Schistosomiasis were updated and issued in 2005, 2011, 2014, 2020, and 2025 by the Chinese Center for Disease Control and Prevention [21,[24], [25], [26]]. These five plans appropriately fused the One Health perspective in the implementation of sentinel surveillance (Fig. 1).

## 5 Development of sentinel surveillance of schistosomiasis in China

### 5.1 First stage: from 1990 to 1998

From 1990 to 1998, the Ministry of Health set 14 fixed villages with a high prevalence of schistosomiasis in eight P/A/M for schistosomiasis surveillance (Table 1). Annual surveys were conducted in these villages to local residents, livestock, and snails to understand the transmission trends and the epidemiological characteristics of schistosomiasis. Also, the results of the surveillance during this period provided evidence for the evaluation of the World Bank Loan Program for the Schistosomiasis Control project implemented in China from 1992 to 2001. The design of the sentinel surveillance in the first stage focused on the infection status of the definitive and intermediate hosts [27]. In 1999, the estimated infection rates were 4.34 % in humans and 4.37 % in cattle [28].

**Table 1:** Distribution and numbers of fixed sentinel surveillance sites from 1990 to 2014 in China.

Provinces/autonomous regions/municipalities	1990–1998	2000–2004	2005–2010	2011–2014
Epidemic area				
Shanghai	1	1	1	1
Jiangsu	1	2	8	7
Zhejiang	0	0	1	1
Anhui	1	2	12	12
Fujian	0	0	0	0
Jiangxi	2	3	12	12
Hubei	3	4	16	16
Hunan	3	4	16	16
Guangdong	0	0	0	1
Guangxi	0	0	0	1
Sichuan	2	4	9	9
Yunnan	1	1	4	4
Non-epidemic area				
Chongqing	0	0	1	1
**Total**	**14**	**21**	**80**	**81**

### 5.2 Second stage: from 2000 to 2004

From 2000 to 2004, the fixed sentinel surveillance sites extended to 21 villages in eight provinces and municipalities (Table 1). Additionally, information reflecting control measures was collected, including chemotherapy for humans and livestock, health education, and mollusciciding [29]. The surveillance results of this period revealed the main challenges of decreasing the morbidity and prevalence of schistosomiasis continuously, such as the limitation of chemotherapy alone to prevent infection and reinfection, the lack of information about the role of cattle in the transmission, and the increased imported cases from high endemic areas to transmission-controlled or transmission-interrupted areas [2]. Based on the recognition of the important role of livestock in the transmission, pilot studies were conducted in lake regions to block the transmission through integrated interventions.

### 5.3 Third stage: from 2005 to 2014

In 2004, the National Medium and Long-term Strategic Plan for Schistosomiasis Prevention and Control (2004–2015) was issued by the Ministry of Health, Finance, Agriculture, and Water Conservancy, the National Development and Reform Commission, and the State Administration of Forestry to accelerate the process of schistosomiasis prevention and control [30]. More efforts were put into the surveillance to evaluate the effect of the new strategy focusing on blocking the transmission of schistosomiasis from reservoir to environments. Besides, the China Center for Disease Control and Prevention took charge of the national surveillance for schistosomiasis since 2005 [21].

**Fig. 2:**
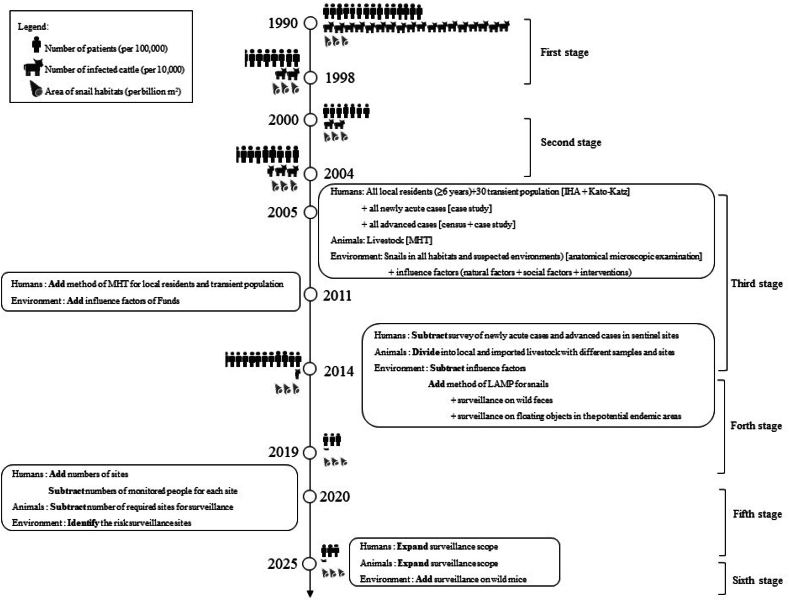
Main changes in the endemic status of schistosomiasis japonica and the content of sentinel surveillance in the four national surveillance plans for schistosomiasis from 1990 to 2025**.** Note: The numbers of patients and infected cattle of schistosomiasis japonica in 1990 are estimated numbers according to the sample survey. Abbreviations: IHA, indirect heamagglutination assay; MHT, miracidium hatching test; LAMP, loop-mediated isothermal amplification.

From 2005 to 2010, 80 villages from 10 P/A/M were selected as the fixed sentinel surveillance sites based on the categories of ecological features and control stages of endemic areas. In this stage, the target population included both local residents and the transient population, while surveillance of livestock was still focused on indigenous domestic animals based on an annual cross-sectional survey after the transmission season. Specific investigations on acute and advanced cases were identified throughout the year in this plan. Besides, natural and social factors with potential influences were also collected, including water level, rainfall, temperature, natural disasters, population mobility, production and lifestyle of residents, etc. (Fig. 2 & Supplementary Table 1)

Great achievements were obtained in schistosomiasis with the amount of cases decreased from 843,000 in 2004 to 326,000 in 2010, with a reduction rate of 61.3 % [31,32]. To further improve the sensitivity and effectiveness, the surveillance plan was revised in 2011 with the adjustment of the fixed sites and modifications in surveillance contents technically [20]: (1) to supplement the Kato-Katz thick smear technique with the miracidium hatching test (MHT) in parallel for individuals who are tested positive for antibodies using the indirect heamagglutination assay (IHA); (2) to increase the sample size of transient population surveillance from 100 to at least 200 in sentinel sites of provinces which had interrupted the transmission of schistosomiasis; (3) to report the newly developed or diagnosed advanced cases only; (4) to conduct stool examination for livestock from one test to three tests per sample; (5) to collect the information of funding that supports control activities as an influencing factor (Fig. 2 & Supplementary Table 1). It should be noted that the non-epidemic area, Chongqing municipality, which was regarded as having the potential transmission risk of schistosomiasis due to the ecological changes caused by the construction and running of the Three Gorges Dam, was also included in the scope of sentinel surveillance [33].

### 5.4 Fourth stage: from 2015 to 2019

After several years of intensive interventions in China, nine endemic P/A/M achieved the criteria of transmission control (prevalence in humans and livestock are both less than 1 %) or even transmission interruption (no new infection occurs in humans, livestock, and snails in five consecutive years) [34]. The infection rates of schistosomiasis decreased to 0.11 % in humans and 0.05 % in cattle in 81 surveillance sites in 2014 [34]. To adapt to the low prevalence setting, another revision of the surveillance plan was issued in late 2014 and implemented since 2015. Fortunately, financial support for national surveillance activities on schistosomiasis was stipulated and included in the central government finance transfer payment program for schistosomiasis since 2014 [25].

A total of 457 villages were set as surveillance sites, covering all endemic counties of 12 endemic P/A/M and four counties in the Three Gorges Reservoir area. The surveillance counties were categorized into four types: counties not achieving the criteria of transmission interruption (Type 1), counties interrupted the transmission of schistosomiasis but still have snails infested (Type 2), counties interrupted the transmission of schistosomiasis without snails distributed (Type 3), and counties in The Three Gorges Reservoir area (Type 4). The main changes in the content of surveillance compared with the previous surveillance plan were as follows: (1) the number of local residents targeted for survey increased but was limited in Type 1 and 2 counties considering the cost-effectiveness and transmission risk; (2) the sample size of monitored livestock increased from “60 of each species” to “a total of 100 for all raised livestock” due to a significant decrease in the number of livestock in endemic villages, and survey on imported livestock was required to be conducted in each Type 2–4 county; (3) snail survey on the floating objects in the water in the Three Gorges Reservoir area was required and a well developed molecular technique named loop-mediated isothermal amplification (LAMP) method combined with sample pooling strategy was recommended to detect the DNA of *S. japonicum* in snails to increase the sensitivity of surveillance by the detection of early infection [[35], [36], [37], [38], [39], [40], [41]].

Additionally, risk surveillance was added as a supplement for early and sensitive identification of risks by snail survey and detection using LAMP method and wild feces surveys in possible high-risk environments. The minimum number of risk surveillance sites was decided by the budget they received from the central government finance transfer payment program for schistosomiasis but could increase according to the local budget and risk situation. The sites for risk assessment were identified as villages with newly discovered snail habitats or infected snails or rebounded infested area in the previous year, and the areas with a relatively high prevalence of schistosomiasis among human and animals, or with dramatic environmental changes caused by natural disasters or engineering construction, or with potential emerging risk of schistosomiasis caused by massive population migration.

### 5.5 Fifth stage: from 2020 to 2024

With the implementation of the national control program, the infection rates of schistosomiasis in humans and cattle gradually decreased to zero in more endemic areas. Till 2019, all 12 P/A/M achieved transmission control criteria, of which five (Shanghai, Guangdong, Guangxi, Fujian, and Zhejiang) achieved elimination and two (Sichuan and Jiangsu) interrupted the transmission of schistosomiasis [42]. To promote the progress of elimination by 2030 and play the pivotal role of surveillance to explore any pocket of infection or risk setting, the surveillance plan was revised again in 2020 [43,44].

Compared with the previous version of the surveillance plan, the main changes were as follows. (1) To identify transmission risk as much as possible, all the sentinel surveillance sites could be mobile, but the number increased significantly to further enlarge the surveillance scope. The specific number and type of risk surveillance sites could contribute to the quality control of the tasks. (2) Surveys on local residents were only required in counties still in the stage of transmission control. The number of surveyed villages increased, but the sample size of residents decreased from 500 to 300 per village, considering the decreased residents in villages and compliance of residents receiving screening. (3) Surveillance on livestock was no longer required in counties of Type 3 and 4 without snails infested at least five consecutive years, but surveillance on imported livestock was required in counties of Type 1. (4) The number of environments for snail survey in risk surveillance was specified.

### 5.6 Sixth stage: from 2025 to present

Till the end of 2023, all the counties achieved the criteria of transmission interruption, which meant the period closest to the elimination. The surveillance plan was again revised to meet the requirements for promoting the process of elimination or consolidating the achievements of elimination considering the zoonotic feature of schistosomiasis japonica and potential transmission that existed in nature. The focus has been transferred to the environment. Thus, to position the risk in the new stage, the surveillance scope on humans and livestock expanded for the surveillance sites, which achieved the criteria of elimination and infestation with snails, to provide the evidence of schistosomiasis elimination in humans and livestock. Besides, the survey on wild mice was added to the content of risk surveillance as supplementary content of the surveillance system.

## 6 Challenges for One Health surveillance when schistosomiasis moves towards elimination

Along with the advancement of the elimination process of schistosomiasis in China, the focus of the national control program moved from snail elimination during the mid-1950s to morbidity control from the mid-1980s to 2003, and then to integrated control focused on infection source control and management from 2004 to the present [45]. Based on previous experiences, there are still several challenges to implementing One Health surveillance strategy in national control programs combating schistosomiasis in China when moving towards elimination.

For humans, the diagnostic tools and techniques of schistosomiasis could not meet the demands of the current low prevalence and infection intensity [46]. The current diagnostic strategy was primary immunodiagnostic screening followed by the Kato-Katz method or MHT for antibody-positive individuals [47]. This serial diagnostic strategy may lead to misdiagnosis, although it is cost-effective when detecting large-scale population. Besides, local residents in elimination communities are not included in the screening, while they may act as the potential infection resources who were missed in the previous examination or were not cured by the pathogenetic treatments [48]. Furthermore, with the increase in international trade and communication, travelers or visitors might carry schistosomes from other endemic countries [49]. Therefore, highly sensitive screening and diagnostic techniques are necessary to enhance the sensitivity and efficiency of the current surveillance system. Additionally, the distribution of the at-risk population has changed due to the current policy of the fishing ban in the Yangtze River basin. The anglers or travelers but not fishermen account for the most of risky population and are difficult to track.

For animals, the low sensitivity of diagnostic tools is also the main challenge. Additionally, surveillance of wild animals, such as goats, canines, deer, and wild mice, are neglected but could be vital in the control of the transmission or consolidation of achievements [50]. A meta-analysis study indicated that the overall pooled infection rate of rodents in the mainland of China was 3.86 % (95 % confidence interval [*CI*]: 2.16 %–5.93 %) [51]. With the replacement of farming livestock by tractors and the removal of bovines, future major reservoir hosts are destined to shift to the wild animals [51]. The wild animals exhibit a large geographic distribution [52] and are hard to monitor, carrying the schistosomes in a large-scale environment for the completion of the life cycle of the parasite.

For the environment, the surveillance system could not cover all the stages of the life cycle of schistosomes. MHT was used to identify the discharged schistosome eggs in the wild feces, but the observation of hatched miracidia by human eyes could lead to missed detection when the amount of eggs in the feces is fairly low. For the miracidia, sporocysts, and cercariae in the intermediate host snails, no infected snails were found by dissection in the surveillance sites, but positive samples of LAMP were reported since 2015 [53], which suggests the high sensitivity of molecular methods. However, not all surveillance sites are required to use LAMP for detection due to its complex operation and high costs. Thus, the potential risk of transmission could be underestimated. Moreover, there are no tools for large-scale detection of the schistosomes in the water environment.

As for the coordination of the whole society, the interaction for the prevention and control of the whole health issue should be recognized. For instance, it is estimated that there will be a mean delay of 2–3 years to achieve the goal of elimination of schistosomiasis by 2030, due to 1-year suspension of control programme of schistosomiasis in areas of high prevalence during the pandemic of COVID-19 [54]. At that time, professionals on schistosomiasis prevention and control were assigned to control the endemic COVID-19. Thus, these potential impacts should be considered, and an advanced plan should be prepared in case of other emergencies. Besides, although the coordination of multiple sectors in schistosomiasis elimination was led by the local governments, how to put the government into a low-risk disease which will be eliminated in recent years remains a big challenge. Additionally, in some areas that faced the challenges of institutional reform and the lack of financial support, the promotion and further consolidation of elimination might be affected.

## 7 How to enhance the One Health surveillance of schistosomiasis

Surveillance represents the final, crucial step in achieving the effective elimination of diseases by providing timely information to monitor disease trends, guide interventions, and evaluate health outcomes [55,56]. Due to the above challenges, more efforts could be put into the objects, approaches, techniques, and multi-sectoral coordination to enhance the current One Health surveillance system of schistosomiasis.

### 7.1 For humans

The diagnostic strategy could be improved by applying novel, highly sensitive techniques and non-species-specific screening tools in routine surveillance. With the development of genomics and genome data for parasites, molecular diagnostic techniques with high sensitivity and specificity and requirement of less equipment and cost were well developed and renewed for future surveillance [57], such as LAMP, recombinase polymerase amplification (RPA), in combination with various result interpretation methods [58]. Also, existing molecular diagnostic methods should be simplified and standardized for large-scale application [59]. Additionally, regular training for these new tools should be held to maintain the detection skill of the laboratorian.

### 7.2 For animals

Sensitive and field-applicable diagnostic procedures for animal reservoirs should be developed. A meta-analysis reviewed research on 14 parasitological, immunological, and molecular techniques and suggested that the parasitological technique formalin-ethyl acetate sedimentation-digestion (FEA-SD) and molecular techniques, especially qPCR, are the most promising techniques with higher sensitivity [60]. Since wild animals are becoming the main animal sources of infection, appropriate strategies should be developed with the cooperation and assistance of departments of agriculture and forestry for the capture and identification of wild animals. Additionally, as the previous effective intervention on livestock showed, the preventive chemotherapy by praziquantel in livestock could be continued to preserve the low transmission [[61], [62], [63]].

### 7.3 For the environment

The emphasis of the surveillance of the environment is the early warning of the transmission risk by identifying the existence of the schistosome in its life cycle before the miracidium attaches to the intermediate snail hosts or cercariae penetrates the skin of the definitive hosts. Firstly, new techniques and methods could be applied in the surveillance from pilot to large-scale areas. For the miracidia hatched from the feces, the dynamic automatic identification system has been developed by machine learning and has been reported with higher efficiency and accuracy for detecting the low-density miracidia [64]. For the parasite in the intermediate host snails, PCR-based parasite DNA detection assays are expensive and are not appropriate for application in large-scale screening with limited funding. Thus, other cost-effective detection techniques and tools should be developed. For the cercariae in the water, several methods have been developed, including sentinel snails or mice, cercarial traps, biomimetic membranes, filtration of water samples, microscopy, and environmental DNA (eDNA) detection [65]. The sentinel mice approach is mostly used in the comprehensive control of schistosomiasis, surveillance of certain major river basins during floodings, and scientific research, but this method is time-consuming [66,67]. Other methods to collect live cercariae in the water body have simple operation procedures but are also limited by the maximum 24 h life expectancy and the low intensity of cercariae in the water body. Aquatic eDNA, in general, consists of nuclear or mitochondrial DNA released from organisms via feces, mucous, gametes, skin, hair, and carcasses and can be detected in the cellular or extracellular form directly in the environment [68]. It persists for a longer time in the environment compared with the short-lived cercariae, which provides new insights for targeting risks related to schistosomiasis.

### 7.4 For the multi-sectoral collaboration

Multi-sectoral collaboration, called for in the One Health approach, has a long history in schistosomiasis control and prevention in China since the 1950s. The effect is significant, although massive manpower and resources are required for the cooperation. For instance, the department of culture and tourism should assist in charge of warning travelers from non-endemic regions to stay away from the high-risk areas with intermediate host snails. The surveillance of animals needs the cooperation among the local department of health, animal husbandry, forestry, and agriculture. The surveillance of the environmental factors, including climate, water, economy, and society, needs further cooperation with meteorological departments and related research institutions. These environmental factors could have an impact on the density of the intermediate snail hosts, which has a significant positive correlation with the infection rate [69,70]. Based on the detailed environmental factors, several statistical models could be developed to predict the real-time transmission risk and the trend of the transmission pattern in different areas [71,72], especially with the utilization of the remote sensing and geographic information system (GIS) technologies [[73], [74], [75]]. Additionally, natural disasters such as flooding and earthquakes also have severe impacts on the transmission of schistosomiasis [69,76,77]. Thus, the early warning of these events is also required. With this support, it is possible to build the multi-point trigger and multi-channel surveillance mechanism in combination with multi-modal data and artificial intelligence for intelligent early warning of infectious diseases. Also, simultaneous surveillance on schistosomiasis and other parasitic diseases could be considered as an approach to save the surveillance costs [78].

## 8 Perspective for further surveillance system in the context of One Health

One Health surveillance is an approach to timely discover, investigate, and eliminate the continuous transmission. When schistosomiasis moves towards elimination of schistosomiasis, new patients, infected livestock, or observed snails with cercaria could be rarely found. Therefore, priority should be given to the surveillance of the environment in the risk surveillance contents. Future surveillance systems should comprehensively control each point in the chain of transmission, and further convenient, sensitive, and inexpensive detection methods should be developed and improved in the diagnosis of lightly infected humans and animals and the surveillance of the existence of schistosomes in the environment for large-scale and point-of-care settings.

## CRediT authorship contribution statement

**Suying Guo:** Writing – original draft. **Lijuan Zhang:** Data curation. **Yifeng Li:** Writing – review & editing. **Shiqing Zhang:** Writing – review & editing. **Xiaojuan Xu:** Writing – review & editing. **Yinlong Li:** Data curation. **Chunli Cao:** Data curation. **Jing Xu:** Writing – review & editing, Funding acquisition, Conceptualization. **Shizhu Li:** Writing – review & editing, Conceptualization.

## Ethics approval and consent to participate

No applicable.

## Consent for publication

No applicable.

## Availability of data and materials

No applicable.

## Funding

This review is funded by National Key Research and Development Program of China (grant numbers 2021YFC2300800, 2021YFC2300804) and National Science Foundation of China (grant number 82073619).

## Declaration of competing interest

The authors declare the following financial interests/personal relationships which may be considered as potential competing interests:Jing Xu reports financial support was provided by National Science Foundation of China. Jing Xu serves as a member of the Editorial Board of *Science in One Health*. To minimize any potential conflicts of interest, she was not involved in the editorial review or decision-making process for this manuscript. If there are other authors, they declare that they have no known competing financial interests or personal relationships that could have appeared to influence the work reported in this paper.
