# Supplementary material for: One Health integrated surveillance: a way forward to accelerate schistosomiasis elimination in China
Source: Sci One Health. 2025 May 16;4:100114. doi: 10.1016/j.soh.2025.100114 (PMC12182346; doi:10.1016/j.soh.2025.100114)
Supplement: Multimedia component 1 [file mmc1.docx]

Supplementary Table 1. Changes in the requirements of sentinel surveillance in the national surveillance plan for schistosomiasis in version 2005, 2011, 2014, 2020 and 2025.

| Sentinel surveillance | Version 2005 (base) | Version 2011 | Version 2014 | Version 2020 | Version 2025 |
| --- | --- | --- | --- | --- | --- |
| 1.1 Regular sentinel surveillance sites | 80 fixed sites in 10 P/A/M^a^ | 81 fixed sites in 12 P/A/M | The sites covered all the 453 endemic counties (13 P/A/M) and 4 counties in The Three Gorges Reservoir area.  Counties not achieving the criteria of transmission interruption (Type 1): 1 fixed site in each county  Counties of transmission interruption with snails (Type 2): 1 fixed site in each county.  Counties of transmission interruption without snails (Type 3): At least 1 mobile site which shift for each year.  The Three Gorges Reservoir area (Type 4): 2 counties in Chongqing and 2 counties in Hubei. | The sites covered all the 451 endemic counties (13 P/A/M) and 4 counties in the Three Gorges Reservoir area.  Counties of Type 1: 3-5 mobile sites in each county where the endemic are more severe based on the data of previous year.  Counties of Type 2: 2-4 mobile sites with severe current snail distribution condition in each county.  Counties of Type 3: 2-4 mobile sites with severe historical snail distribution condition and high risk of snail importation or with more suitable environments for habitats in each county.  Counties of Type 4: Same as version 2014. | The sites covered all the 450 endemic counties (13 P/A/M) and 4 counties in the Three Gorges Reservoir area.  Counties of Type 1: 3 fixed sites in each county.  Counties of Type 2: 1 fixed site and 2 mobile sites with current snail distribution condition in each county.  Counties of Type 3: 3 mobile sites with severe historical snail distribution condition and high risk of snail importation or with more suitable environments for habitats in each county.  Counties of Type 4: Same as version 2014. |
| 1.2 Risk surveillance sites | None. | None. | Several villages or certain regions are identified as risk surveillance sites. The number was not confined.  The selection criteria of sites: Newly snail habitats, or environment with infected snails, or region with rebounded snail area and density in the previous year; or areas with high prevalence of human and animal schistosomiasis; or regions with environment change by natural disasters or engineering construction, or regions where massive population migration could lead to schistosomiasis endemic. | All Type 1 counties: 3 sites in each county.  No less than 20% of Type 2 counties: 2 sites in each county.  The selection criteria of sites are same as version 2014. | Same as version 2020. |
| 2 Content | | | | |  |
| 2.1 Human |  |  |  |  |  |
| 2.1.1 Local residents | All the local residents over 6 years old in each site should be examined by IHA^b^. People with positive IHA results should be further examined by Kato-Katz. | Added miracidium hatch test (MHT) for the individuals with positive IHA results. | Type 1: No less than 500.  Type 2: No less than 300.  Others: No requirements. | Type 1: No less than 300.  Others: No requirements. | Type 1 for each year and type 2 for 2025, 2027, and 2029: No less than 300.  Others: No requirements. |
| 2.1.2 Transient population | 7 P/A/M (Hunan, Hubei, Jiangxi, Anhui, Jiangsu, Sichuan, and Yunnan): 30 transient people in each site are randomly selected.  3 P/A/M (Zhejiang, Shanghai, and Chongqing): 100 transient people in each town where the sites located.  The examination methods are the same as 2.1.1. | 5 P/A/M (Zhejiang, Shanghai, Chongqing, Guangdong, and Guangxi): No less than 200 in each town where the sites located.  Added MHT for the individuals with positive IHA results. | Type 1-4: No less than 200 in each county.  Risk surveillance sites: No requirements. | Same as version 2014. | Same as version 2014. |
| 2.1.3 Acute cases | Case survey for all the acute cases in the surveillance year. | Same as version 2005. | None. | None. | None. |
| 2.1.4 Advanced cases | In the first year, a census should be done among all the advanced cases in the surveillance sites. From the second year, examination on suspected advanced cases and case survey on newly advanced cases should be conducted. | Only case survey on newly and existed advanced cases. | None. | None. | None. |
| 2.2 Livestock | 60 of each species of livestock, such as cattle, sheep, pigs, and horses, in each site should be examined by MHT. If less than 60, examine all. | The observation frequency of MHT increased from one to three parallel observation by three operators for less personal error. | Type 1: No less than a total of 100 livestock.  Type 2-4: No less than a total of 100 imported livestock.  Risk surveillance sites: No requirements. | Type 1: No less than 100 local livestock and no less than 100 imported livestock.  Type 2: No less than 100 imported livestock.  Others: No requirements. | Type 1 and others: Same as version 2020.  Type 2: No less than 100 local livestock in 2025, 2027, and 2029; and no less than 100 imported livestock for each year. |
| 2.3 Environment |  |  |  |  |  |
| 2.3.1 *Oncomalania hupensis* | Snail survey should be done in the existing snail habitats (including susceptible environment and other snail-infested environment) and suspicious environment. | The scope was defined more specifically as “all the existing snail habitats and suspicious environment in the major living environments in the local village and surrounding areas”.  The suspicious environment was specifically defined as the environments adjacent to or connected by hydrographic net with the current existing snail habitats. | Added loop-mediated isothermal amplification (LAMP) as the method to detect the *S. japonicum* infection of snails in Type 1 counties. Other types should also conduct LAMP if have appropriate conditions.  Type 3: Snail survey in the historical snail habitats, the zone of the territorial border of historical snail habitats and snail-free areas, and the environments with high risk of snail importation.  Type 4: more than 5 suspicious environments with risk of snail importation.  Others: Same as version 2011. | Type 1-3: All the existing snail habitats, historical snail habitats, and suspicious environments.  Type 4: No requirements.  Risk surveillance sites: More than 5 snail habitats in each site. The surveyed snail frames should be more than 200 and collected living snails should be more than 500. | Same as version 2020. |
| 2.3.2 Floating object in the water | None. | None. | Type 4: finding *Oncomalania* on the floating objects in more than 5 waters of the Yangtze River.  Others: No requirements. | Same as version 2014. | Same as version 2014. |
| 2.3.3 Wild feces | None. | None. | Type 1 and risk surveillance sites: Wild feces should be collected in the wild environments with frequent activities of human and livestock, and the areas of snail surveillance. No less than 100 wild feces should be collected and examined by miracidia hatching technique.  Type 2-4: No requirements. | Risk surveillance sites: The method is same as version 2014.  Others: No requirements. | Same as version 2020. |
| 2.3.4 Influence factors | (1) Natural and social factors: including water level, rainfall, temperature, natural disasters, population mobility, production and life style, etc.  (2) The implementation of prevention and control: including snail survey, mollusuicide, environment modification, infection investigation, treatment (chemotherapy), health education, personal protection, and improvement of drinking water and lavatories, etc. | Added the information of funding input: including the funding for diseases investigation and treatment, snail survey and mollusuicide. | Data collection of influence factors variables was included in other schistosomiasis control programs. | Data collection of influence factors variables was included in other schistosomiasis control programs. |  |
| 2.3.5 Wild mice | None. | None. | None. | None. | Survey of wild mice in at least 2 snail habitats or possible snail habitats where humans and animals could reach should be conducted in each risk surveillance site. |

^a^ P/A/M: provinces/ autonomous regions/ municipalities0

^b^ IHA: indirect heamagglutination assay
